# Hypoxia-Inducible Factor Stabilizers in End Stage Kidney Disease: “Can the Promise Be Kept?”

**DOI:** 10.3390/ijms222212590

**Published:** 2021-11-22

**Authors:** Giuseppina Crugliano, Raffaele Serra, Nicola Ielapi, Yuri Battaglia, Giuseppe Coppolino, Davide Bolignano, Umberto Marcello Bracale, Antonio Pisani, Teresa Faga, Ashour Michael, Michele Provenzano, Michele Andreucci

**Affiliations:** 1 Department of Health Sciences, “Magna Graecia” University, I-88100 Catanzaro, Italy; giusycrugliano2@gmail.com (G.C.); gcoppolino@unicz.it (G.C.); davide.bolignano@gmail.com (D.B.); teresa_faga@yahoo.it (T.F.); ashourmichael@yahoo.com (A.M.); 2 Department of Medical and Surgical Sciences, University Magna Graecia of Catanzaro, Viale Europa, I-88100 Catanzaro, Italy; rserra@unicz.it; 3 Interuniversity Center of Phlebolymphology (CIFL), “Magna Graecia” University, I-88100 Catanzaro, Italy; 4 Department of Public Health and Infectious Disease, “Sapienza” University of Rome, I-00185 Roma, Italy; nicola.ielapi@uniroma1.it; 5 Division of Nephrology and Dialysis, St. Anna University-Hospital, I-44121 Ferrara, Italy; battagliayuri@gmail.com; 6 Vascular Surgery Unit, Department of Public Health, University Federico II of Naples, I-80131 Naples, Italy; umbertomarcello.bracale@unina.it; 7 Department of Public Health, University Federico II of Naples, I-80131 Naples, Italy; antonio.pisani13@gmail.com

**Keywords:** chronic kidney disease, anemia, erythropoietin, treatment, renal disease, renal failure, ESAs

## Abstract

Anemia is a common complication of chronic kidney disease (CKD). The prevalence of anemia in CKD strongly increases as the estimated Glomerular Filtration Rate (eGFR) decreases. The pathophysiology of anemia in CKD is complex. The main causes are erythropoietin (EPO) deficiency and functional iron deficiency (FID). The administration of injectable preparations of recombinant erythropoiesis-stimulating agents (ESAs), especially epoetin and darbepoetin, coupled with oral or intravenous(iv) iron supplementation, is the current treatment for anemia in CKD for both dialysis and non-dialysis patients. This approach reduces patients’ dependence on transfusion, ensuring the achievement of optimal hemoglobin target levels. However, there is still no evidence that treating anemia with ESAs can significantly reduce the risk of cardiovascular events. Meanwhile, iv iron supplementation causes an increased risk of allergic reactions, gastrointestinal side effects, infection, and cardiovascular events. Currently, there are no studies defining the best strategy for using ESAs to minimize possible risks. One class of agents under evaluation, known as prolyl hydroxylase inhibitors (PHIs), acts to stabilize hypoxia-inducible factor (HIF) by inhibiting prolyl hydroxylase (PH) enzymes. Several randomized controlled trials showed that HIF-PHIs are almost comparable to ESAs. In the era of personalized medicine, it is possible to envisage and investigate specific contexts of the application of HIF stabilizers based on the individual risk profile and mechanism of action.

## 1. Introduction

Anemia is a common complication of chronic kidney disease (CKD). The presence of anemia in patients with CKD is associated with increased risk for hospitalization, cognitive impairment, reduced quality of life, and major cardiovascular events. Furthermore, the severity of anemia is an independent predictor of mortality [1]. As recently shown, the prevalence of anemia as well as that of each metabolic complication in CKD strongly increases as the estimated Glomerular Filtration Rate (eGFR) decreases. Moranne et al. [2] observed that as mGFR decreased from 60 to 90 to < 20 mL/min per 1.73 m^2^, the prevalence of anemia increased from 8–41% (the analysis using eGFR also produced similar results). Although anemia and hyperparathyroidism were observed early in stage 3 CKD, other disorders (acidosis, hyperkalemia, and hyperphosphatemia) appeared later in the course of the disease [2]. Many other studies found that diabetic nephropathy is related to a higher risk of anemia, independently of eGFR levels [2,3,4]. Moreover, obesity has been associated with a lower risk for anemia onset. Mechanisms such as better nutrition and less iron deficiency in obese patients, compared with normal-weight patients may partially explain this pattern. Based on this evidence, it has been postulated that testing for anemia should be started early, during stage 3 of CKD, or even earlier in patients with diabetes [2]. The early onset of anemia in CKD patients has a pathophysiologic explanation. At the initial stage, several conditions directly associated with CKD, such as chronic inflammation and iron deficiency, cause EPO resistance, whereas in the more advanced stage of CKD, both EPO resistance and EPO deficiency develop [5]. For these reasons, a huge number of CKD patients do not respond to treatment with erythropoiesis-stimulating agents (ESAs) alone. The prevalence of ESA hypo-responsiveness was quantified in previous studies and ranges between 12% and 30%, depending on the definition criteria [6]. Moreover, ESA hypo-responsiveness is associated with increased risk for all-cause mortality, cardiovascular events, and CKD progression.

Hence, the appropriate treatment of anemia in CKD patients is critical, since it may help to reduce risk for the onset of all these events. To this end, a novel drug class that has been shown to be effective at treating anemia in CKD patients and with different mechanisms of action has been developed and may offer wide applications in the future. The objective of the present review is to describe the evidence derived from some randomized trials that tested both the efficacy and safety of HIF stabilizers, particularly *Roxadustat* and *Vadadustat*, in end-stage kidney disease (ESKD) compared to the standard of care. The mechanism of function and regulation of the HIF system is also briefly analyzed, as well as the current management of anemia in CKD/ESKD. The review concludes with a hypothesis as to the possible applications of HIF stabilizers in the context of personalized medicine.

## 2. Anemia in Chronic Kidney Disease

The pathophysiology of anemia in CKD is complex. Its main causes are EPO deficiency and functional iron deficiency (FID) [7]. Other causes include uremia, a condition where hemolysis is common due to increased red blood cell deformity and, in hemodialysis patients, due to small but frequent losses of blood. 

### 2.1. EPO Deficiency

The main cause of renal anemia is renal damage, which affects the secretion of EPO [8]. Under normal circumstances, the main stimulus for transcription of the EPO gene is tissue hypoxia, which can cause a rapid increase in EPO blood levels. Hypoxia is generally associated with anemia and induces the transcription of HIF-1α (hypoxia inducible factor-1α) in renal peritubular interstitial cells, where it binds to the subunit HIF-1β, which is a constitutively expressed subunit. This assemblage promotes the transcription of target genes, including the EPO gene. After binding with EPO, the erythropoietin receptor (EPOR) forms a homodimer that promotes cell proliferation and prevents cell apoptosis. In the bone marrow, EPO stimulates the survival and proliferation of erythroid precursors and induces their differentiation into mature erythrocytes [8]. The consequent gain in hematocrit increases the blood’s ability to transport oxygen [9]. Finally, it is interesting to remember that EPO exerts several effects, besides those on bone marrow cells, in different tissues and organs, since its receptor has been found in several cell types; in fact, EPO, in its different forms (i.e., alpha, darbepoetin), can exert opposite (protective or detrimental) effects in several injury models, including those studied in renal cells [10,11,12,13,14,15,16,17]. 

### 2.2. Functional Iron Deficiency

Several elements are responsible for maintaining iron homeostasis. The main source of iron for erythropoiesis comes from the iron released by macrophages. These reticuloendothelial cells swallow red blood cells to reabsorb iron in hemoglobin and transport it to serum with ferroportin (FPN) [8]. At the same time, divalent metal transporter 1 (DMT1) and duodenal cytochrome b (DcytB) are both important components of the intestinal absorption of iron, as they both contribute to the absorption of iron in food and to then transport it into duodenal cells [8,9,18]. Even in this mechanism, iron is carried out of intestinal cells and into the blood through FPN [9]. This implies that iron absorption is closely related to the transmembrane expression of FPN in enterocytes. Another important regulator of iron metabolism is hepcidin, a peptide hormone produced by the liver, which, by binding FPN, promotes its internalization and degradation, thus preventing the absorption of iron from the small intestine and macrophages.

Chronic Kidney Disease is considered an inflammatory disease [19]. Inflammatory anemia is a common feature in patients with advanced CKD and an established risk factor for ESKD [20]. Inflammation has been shown to promote increased hepatic secretion of hepcidin through the interleukin 6 (IL-6)—STAT3 pathway [21,22] and the bone morphogenetic protein (BMP)—SMAD pathway [23]. Furthermore, it has been demonstrated that hepcidin can also inhibit the secretion of DMT1 and DcyB [24,25]. In this way, inflammation interferes with hematopoiesis; thus, hepcidin is responsible for anemia and ESA resistance [26]. In addition, the oxidative stress related to inflammation leads to lipid peroxidation of erythrocyte membrane [27] and, consequently, to a shorter red blood cell lifespan [28]. 

### 2.3. HIF Regulation in CKD

It has been demonstrated that the cellular hypoxia response mediated by the HIF pathway is finely regulated [29]. The prolyl-4-hydroxylase domain enzymes (PHDs) hydroxylate HIF-α under normoxia, leading to an increased affinity with the von Hippel Lindau (VHL) protein. The VHL protein is part of the complex that targets HIF-α for proteasomal degradation [30]. Under the same conditions, another regulatory enzyme, asparagine hydroxylase factor inhibiting HIF (FIH), prevents the interaction between the acetyltransferase p300/CBP and the HIF heterodimer, thus reducing HIF activity. Conversely, following hypoxic stimuli, HIF-α is stabilized and activated by p300/CBP; it acts by enhancing the expression of target genes. However, in the specific context of CKD, these mechanisms are even more complex. In fact, PHDs, which can be considered sensors for hypoxia, also activate NF-ĸB via the IĸB kinase-β (IKK,) which in turn phosphorylates and degrades IĸB. Even more importantly, hypoxia, other than activating NF-ĸB, elicits several pro-inflammatory patterns triggered by NF-ĸB, such as leucocyte activation and cytokine production. These mechanisms are even reinforced in the presence of CKD given that CKD is considered an inflammatory disease per se [31] and, at least in part, explain the multiple connections between hypoxia, inflammation, and CKD progression. In experimental and clinical studies in humans, it has been shown that hypoxia stimulates inflammation and that in turn inflammatory lesions become hypoxic, perpetuating a vicious circle over time [32]. 

Recent studies have shown that the HIF pathway is downregulated in CKD and this has been attributed to a reduced renal microvasculature, a lack of Vascular Endothelial Growth Factor expression in the kidney, and increased tubular atrophy [33,34]. Moreover, the pharmacological stimulation on EPO itself by HIF stabilizers may indicate the suboptimal activation of the HIF pathway in CKD patients. 

## 3. Management of Anemia in CKD

**Figure 1:**
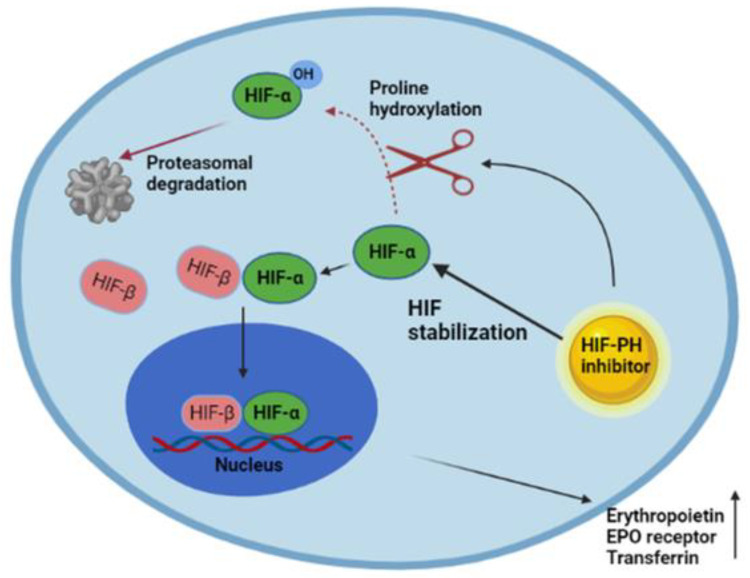
Mechanism of action of HIF stabilizers. Under normal conditions, the activity of HIF-PH leads to the rapid degradation of HIF. During hypoxia, HIF-PH activity is instead suppressed, resulting in stimulation of endogenous EPO production, increased expression of the transferrin receptor, better use of iron with consequent maturation of erythrocytes, and an increase in hemoglobin levels. Similarly, the use of HIF stabilizers results in a steady increase in dose-dependent hemoglobin levels, including reducing hepcidin and ferritin levels and improving iron binding capacity.

The administration of injectable preparations of recombinant ESAs, especially epoetin and darbepoetin, coupled with oral or iv iron supplementation, is the current mainstay of treatment for anemia in CKD for dialysis (DD-CKD) and non-dialysis (NDD-CKD) patients [1,35]. This approach reduces patients’ dependence on transfusions, ensuring the achievement of optimal hemoglobin target levels. However, there is still no evidence that treating anemia with ESAs can significantly reduce the risk of cardiovascular events [36]. Patients who require very high ESA doses to reach the hemoglobin target have an increased mortality and hospitalization rates [1]. Meanwhile, iv iron supplementation can expose a patient to an increased risk of allergic reactions, gastrointestinal side effects, infection and cardiovascular events [37,38]. The KDOQI guidelines suggest that iron supplementation should be performed in order to maintain ferritin levels > 200 ng/mL in hemodialysis patients and >100 ng/mL in peritoneal dialysis patients and non-dialysis patients with CKD; in all these categories, the transferrin saturation (TSAT) must be kept above 20%. Iron supplementation is not recommended when the TSAT is > 30% and ferritin > 500 ng/mL. The CHOIR trial showed that patients with CKD had a higher risk of a composite end point that included death and cardiovascular events if they were treated to a hemoglobin target level of 13.5 g/dL rather than 11.3 g/dL [39], and the TREAT trial showed that patients with diabetes mellitus and CKD treated with darbepoetin alfa (to a target hemoglobin level of 13 g/dL) had a higher risk of stroke than those who received a placebo; in addition, they experienced only a minimal improvement in fatigue [40,41]. This evidence, confirmed in several studies, has led the authorities to recommend the prescription of the lowest ESA dose adequate for reducing the need for red blood cell transfusions [42]. Even more importantly, currently there are no studies defining the best strategy in using ESAs to minimize possible risks of cardiovascular events [43]. Another important point is that of hypo-responsiveness to erythropoietin. The National Kidney Foundation Kidney Disease Outcomes Quality Initiative (NKF KDOQI) defined hypo-responsiveness to EPO as the presence of at least one of the following conditions: (1) a significant decrease in hemoglobin level at a constant EPO dose, (2) a significant increase in the EPO dose required to preserve a certain hemoglobin level, or (3) a failure to raise the hemoglobin level to >11 g/dL despite an EPO dose equivalent to EPO >500 IU/kg/week [44]. In this context, the molecular mechanism that regulates the hypoxic induction of erythropoiesis has been identified as a possible solution (Figure 1).

## 4. Use of HIF Stabilizers in ESKD

One class of agents under evaluation acts to stabilize hypoxia-inducible factor (HIF) by inhibiting prolyl hydroxylase (PH) enzymes [7]. Several randomized controlled trials showed that HIF-PHIs are almost comparable to ESAs [35].

### 4.1. Roxadustat

*Roxadustat* (also known as FG-4592) is a second-generation small-molecule HIF-PH inhibitor that stabilizes HIF-alfa subunits and prevents their degradation by mimicking a hypoxic state, which results in increased HIF transcriptional activity [45]. This leads to the functional activation of early response target genes encoding proteins such as EPO, EPOR, enzymes of heme biosynthesis, and proteins that promote iron absorption and transport (Figure 1) [7,45]. Such mechanisms allow for a physiological level of EPO to stimulate red blood cell production rather than the high intermittent blood level that results from the pharmacological administration of an exogenous ESA [46]. *Roxadustat* is an orally administered molecule which has a half-life of approximately 12 to 15 h and is primarily metabolized by phase I oxidation via cytochrome P450 (CYP)2C8, and phase II conjugation via UDP-glucuronosyltransferase 1-9 (UGT1A9) [47,48,49,50].

Meta-analyses of randomized controlled trials suggest that *Roxadustat* can improve hemoglobin plasma levels and iron metabolism in both DD-CKD and NDD-CKD patients [51,52].

Many studies, including that of Besarab et al. [53], showed that oral *Roxadustat* administered BIW or TIW (respectively two or three times a week) in NDD-CKD patients increased hemoglobin levels in the absence of iv iron supplementation. Functional iron deficiency is a common cause of suboptimal hemoglobin response to EPO analogue therapy [54]. For this reason, iv iron supplementation is required in ESA therapy. Numerous investigations demonstrate that HIF acts as an iron sensor and its stabilization is associated with hepcidin suppression, increased intestinal iron absorption, and increases in iron transport enzyme [55]. Zheng et al. [45], in their meta-analysis, provide evidence for the efficacy and safety of *Roxadustat* for anemia treatment in patients with CKD. The results showed that for NDD patients, both hemoglobin and transferrin blood levels were significantly increased in the *Roxadustat* group versus those in the placebo group, and hepcidin, ferritin and TSAT blood levels were significantly reduced in the *Roxadustat* group versus those found in the placebo group. 

Among the studies conducted in hemodialysis patients, Provenzano et al. [42] and Besarab et al. [56] are among the most interesting. In particular, Besarab et al. [56] first reported hemoglobin correction with an oral HIF-PHI in erythropoietin analogue-naive anemic incident dialysis patients. The HIF-PHI *Roxadustat* increased the mean hemoglobin concentration by ≥2 g/dL within 7 weeks of treatment, independently of baseline hemoglobin levels, iron-repletion status, iron supplementation regimen, inflammatory status, and dialysis modality, while it reduced hepcidin blood levels, thus increasing iron availability for erythropoiesis. These points have been confirmed by Chen et al. [57]. This phase 3 trial showed a statistically significant non-inferiority increase in hemoglobin blood levels with *Roxadustat* relative to epoetin alfa. In contrast with ESAs, *Roxadustat* treatment promotes a physiological and modest endogenous erythropoietin level increase, while concomitantly addressing iron availability with oral iron alone. *Roxadustat* may obviate or reduce the requirement for iv iron, potentially reducing the exposure to the iv iron safety liabilities. Based on these results and those of historical ESA phase 3 studies, *Roxadustat* appears to be effective and comparable to epoetin alfa in maintaining hemoglobin blood levels in patients with ESKD receiving hemodialysis. Finally, many studies have demonstrated that the *Roxadustat*-induced hemoglobin response is independent of baseline C-reactive protein (CRP) blood levels. This suggests that *Roxadustat* may be effective in some patients, even when the inflammatory component of CKD is present [58,59]. Inflammation increases an ESKD patient’s dose requirements for epoetin alfa, whether during correction or maintenance. Resistance to ESAs appears to be mediated in part by elevated hepcidin blood levels [60,61], which are increased in inflammation, limiting iron availability. 

Observations from these studies suggest the effect of *Roxadustat* on serum cholesterol levels. This effect was independent of statin use and could be mediated, in part, by the effects of HIF on degradation of 3-hydroxyl-3methylglutaryl coenzyme A reductase. Reductions in cholesterol blood levels have been reported during high altitude exposure [62].

In conclusion, the HIF oxygen-sensing pathway has been confirmed as a crucial pathway in maintaining hemostasis [63]. The significant induction of TIBC and the reduction of TSAT and ferritin values after treatment with *Roxadustat* indicated the enhanced iron utilization of NDD-CKD patients. These changes in iron metabolism are not only regulated by the interaction between hepcidin and HIF, but also by other iron-related proteins, such as transferrin receptor, DMT1, and ferroportin 1, among others [64]. HIF could directly bind to the hypoxia responsive mRNA regions to regulate the expression of iron-related proteins [65]. When it comes to the results of DD-CKD patients, no significant changes in ferritin and hepcidin blood levels were found after treatment with *Roxadustat*. HIF stabilizer may be less effective for DD- CKD patients than for NDD-CKD patients. The underlying mechanism is unknown. Heterogeneities were evident for plasma levels of hepcidin and ferritin. 

Finally, in June 2021 the results of the *DOLOMITES* study [66] were disclosed. This is a randomized, open-label, phase 3 study that compares the anemia control efficacy and safety of *Roxadustat* versus darbepoetin in 616 NDD-CKD patients. It was demonstrated that *Roxadustat* is a viable option to treat anemia in NDD-CKD patients, maintaining hemoglobin plasma levels for up to 104 weeks. Hemoglobin response to *Roxadustat* administration was not lower than that of darbepoetin alfa administration. *Roxadustat* was not inferior to darbepoetin alfa for changes in mean arterial pressure and the time to occurrence of hypertension; it was superior for changes in LDL levels and for the time of the first intravenous iron use. The safety profiles were comparable between the two groups and there was no difference between the groups regarding the composite endpoints, major adverse cardiovascular events (MACE). 

The data from trials illustrate that *Roxadustat* corrects anemia in CKD patients with some distinct advantages [37]. One of the most important advantages is that *Roxadustat* could induce the transient activation of HIF and also increase the expression of HIF-regulated genes. Its half-life of approximately 12 h enables HIF transcriptional activity to return to the baseline between doses, which results in the induction of EPO expression in a titratable manner [37,67]. Furthermore, *Roxadustat* transiently increased endogenous EPO plasma levels within or near the physiological range in patients with anemia due to CKD. It appears that anemia correction with *Roxadustat* could avoid the potential adverse effects caused by high ESA doses [37]. Beyond elevating EPO plasma levels, *Roxadustat* could correct the degree of anemia by handling iron metabolism and particularly by decreasing hepcidin plasma levels, a response that is independent of the baseline CRP plasma levels and disease state of the CKD patient. This allows the improvement of functional iron deficiency, as well as the increased absorption of oral iron, thus avoiding the use of high doses of iv iron, which has been associated with an increased inflammatory state and increased mortality [68]. The increase in transferrin induced by *Roxadustat* is a direct effect of HIF activation, as there are two HIF binding sites in the gene encoding transferrin [69]. A summary of phase 2 and phase 3 trials with *Roxadustat* is reported in Table 1 and Table 2, respectively.

Encouraging findings are also available regarding the safety of this drug. Zheng et al. [35] showed that for non-dialysis patients, the proportion of all-cause mortality in different HIF-PHIs, EPO, and DPO patient groups was comparable to that of subjects treated with the placebo. Similarly, a previous meta-analysis [71] did not find any difference in adverse reactions when HIF-PHIs were compared with a placebo. Given the case of fatal hepatitis that occurred with FG-2216, liver function was closely monitored: there was no evidence of liver toxicity or sustained increases of liver enzymes or serum bilirubin [36]. Some clinical trials [70,72] have found that HIF-PHIs, such as *Roxadustat*, lower the total cholesterol plasma levels. Considering that HIF-PHIs are involved in different pathways, possibly with increased relevant side effects, their safety must be proven by large, long-term studies [35]. Accruing evidence has shown that HIF transcription factor directly regulates hundreds of genes, and consequently plays an important role in a broad spectrum of cellular functions and biological processes other than erythropoiesis, including energy metabolism, angiogenesis, mitochondrial metabolism, cellular growth and differentiation, inflammation, cell motility, matrix production, and epigenetics [37,73]. For example, HIF-PHIs may influence tumor growth, because HIF activation in hypoxic environments may help already existing tumors to survive and expand [7]. Hypertension is a possible side effect occurring with ESA therapy; only a minority of patients treated with *Roxadustat* reported a worsening in blood pressure control [22]. Phase 3 trials demonstrated a slightly higher risk of hyperkalemia with *Roxadustat* than with a control (epoietin alfa or placebo). In addition, the incidence of metabolic acidosis that was reported as an adverse event was higher in the *Roxadustat* group than the one in the control group (placebo) [37,74]. Furthermore, a potential proangiogenic effect related to the vascular endothelial growth factor (VEGF) and VEGF receptors, the possibility of development of pulmonary hypertension, and the potential progression of kidney disease related to HIF activation over long periods, are three interesting questions [37,73]. None of the ongoing phase 3 clinical trials have been interrupted, so far, for safety issues [36].

### 4.2. Vadadustat

*Vadadustat* (also known as AKB-6548) is an orally administered HIF-PHI that inhibits all three PHDs (with a preference for PHD3) and stabilizes both HIF-1α and HIF-2α [75,76]. *Vadadustat* was approved in Japan on June 2020 for the treatment of adult patients with anemia secondary to CKD. Regular submissions are planned in the USA and the EU [77]. To briefly examine *Vadadustat* in terms of its efficacy and clinical benefits, we discuss two important trials: *INNO2VATE* and *PRO2TECT*. The *INNO2VATE* trials comprise two phase 3, open-label, sponsor-blind, active-controlled trials designed to evaluate the safety and the efficacy of *Vadadustat* versus the ESA darbepoetin alfa in ameliorating anemia in patients with DD-CKD. The study periods include correction/conversion (weeks 0–23), maintenance (weeks 24–52), long-term treatment (weeks 53 to end of treatment) and safety follow up. The primary safety endpoint was the time of the first major adverse cardiovascular event, whilst the primary efficacy endpoint was the change in hemoglobin (baseline to weeks 24–36) [78]. *Vadadustat* was not inferior to darbepoetin alfa in terms of its effects on hemoglobin plasma levels and cardiovascular safety outcomes. *INNO2VATE* correction/conversion enrolled incident dialysis patients, who had initiated peritoneal dialysis or hemodialysis ≤ 16 weeks prior to screening and had limited exposure to recombinant ESAs. The mean hemoglobin plasma level between weeks 24 to 36 was 10.36 g/dL in the *Vadadustat*-treated patients, compared with 10.61 g/dL in the darbepoetin alfa group. The mean hemoglobin plasma level between weeks 40 to 52 was 10.51 g/dL in the *Vadadustat* group versus 10.55 g/dL in the darbepoetin alfa recipients [78]. *INNO2VATE* conversion enrolled dialysis patients currently receiving recombinant ESAs, who were then converted to either *Vadadustat* or darbepoietin alfa. The mean hemoglobin plasma levels between weeks 24 to 36 were 10.36 and 10.53 g/dL in the *Vadadustat* and the darbepoetin alfa recipients, respectively. The mean hemoglobin plasma levels during weeks 40–52 were 10.40 g/dL and 10.58 g/dL in the *Vadadustat* and darbepoetin alfa groups, respectively [69]. In conclusion, in a combined analysis of data from both *INNO2VATE* trials *Vadadustat* was not inferior to darbepoetin alfa in the time to the first MACE event and in correction and maintenance of hemoglobin concentration in patients with CKD who were undergoing dialysis [79]. In both trials, the mean serum concentration of hepcidin and ferritin and the main transferrin saturation were similar in the *Vadadustat* and darbepoetin alfa groups during weeks 24 to 36 and weeks 40 to 52. 

The phase 3 *PRO2TECT* trial [80] in NDD-CKD comprises two studies: *PRO2TECT* correction in patients not currently being treated with ESAs and *PRO2TECT* conversion in patients currently receiving recombinant ESAs who were switched to either *Vadadustat* or an active control. It was shown that there was no difference in the hemoglobin levels achieved, which substantially overlapped in the two groups (darbepoetin vs. *Vadadustat*). Surprisingly, a higher incidence of MACE was found in patients treated with *Vadadustat* compared with those treated with darbepoetin. In conclusion, in these two trials it was found that, among patients with NDD-CKD, *Vadadustat* was not inferior to darbepoetin alfa with regard to hematologic efficacy but did not meet the pre-specified non-inferiority criteria for cardiovascular safety, which were: a composite of death from any cause, non-fatal myocardial infarction, or non-fatal stroke. 

### 4.3. Other Drug Agents

Other molecules currently undergoing clinical trials are *Daprodustat*, *Molidustat*, *Enarodustat*. These drugs are being demonstrated in phase 2 and 3 studies to improve hemoglobin plasma levels and iron metabolism in CKD.

## 5. HIF Stabilizers in Personalized Medicine Era 

The issue of whether ESA can be fully or partially replaced by HIF stabilizer in future treatment is worth debating, but at this point, there are no appropriate guidelines, except for the recommendation released by the Japanese Society of Nephrology (JSN) on 29 September 2020 [81]. In an era of personalized medicine, it is possible to consider specific contexts for the clinical application of HIF stabilizers based on the individual risk profile and mechanism of action. In recent years, research has moved away from traditional protocols and towards so-called “*Precision Medicine*”. According to the National Institutes of Health (NIH), *Precision Medicine* is an emerging approach to disease treatment and prevention that evaluates the variability of each person’s genes, environment, and lifestyle. It is a different concept to that of “one size fits all”. This approach allows researchers and doctors to predict which treatment, in terms of cure and prevention, will work in a specific group of patients [82,83,84]. The application of *Precision Medicine*, initially focused on oncology, is now quite broad. The hope is that the use of genetic and molecular information will become an integral part of medical practice. Therefore, research is changing from classic clinical trials towards study designs that can significantly reduce the variability of the individual response to treatment and optimize the individual prognosis [85,86]. In this context, *Umbrella*, *Basket*, and *Platform* trials are emerging. In *Basket trials*, the effectiveness of a target treatment is assessed in multiple pathologies that have a similar etiology and similar pathophysiological mechanisms. *Umbrella trials* test different drugs for a single disease by stratifying the study population into subgroups on the basis of molecular alterations (biomarkers). *Platform trials* aim to identify, by evaluating multiple treatments at the same time, the therapy for a subgroup of patients selected from a heterogeneous population based on histological data, a biomarker, a risk factor, and a clinical variable (such as age, sex, etc.). It is possible to consider an HIF stabilizer in clinical situations in which there is an obvious presence of an ESA-resistant anemia or of ESA-unresponsiveness or an MIA syndrome and of an iron-dysregulated state, especially at the dialysis stage. The determination of the patient’s iron status and the evaluation of the possible causes of ESA resistance are crucial for the choice of HIF stabilizer. In addition to these indications, consideration should be to the prevention of adverse events due to the use of HIF stabilizers’. Most clinical adverse events do not have completely clear causes. These adverse events include thrombosis/embolism, relapse of latent cancer, and worsening of pulmonary hypertension [81]. It is not currently possible to draw any scientific conclusions on this point, because the length of the observation period in most studies is too short.

## 6. Conclusions

In conclusion, HIF stabilizers have been shown to correct anemia and to help to maintain hemoglobin concentration levels over time in a wide range of levels of CKD severity, including ESKD. Moreover, some studies have also provided evidence of the non-inferiority of the effect of HIF stabilizers compared with the that of injectable EPO therapies with respect to the development of major cardiovascular events. Future studies are needed to understand which patients are likely to respond better to these drugs and whether these novel treatments can replace the standard treatment with injectable ESAs.

## Figures and Tables

**Table 1:** Phase 2 studies of Roxadustat.

First Author	Patients	Study	Randomization	Primary Endpoint	Follow-Up	Results
Besarab, 2015 [53]	117 NDD-CKD	Correction	3:1 (placebo)	Change in Hb from baseline and proportion of Hb responders (Change in Hb ≥ 1.0 g/dL)	4 weeks	Roxadustat significantly increased endogenous erythropoietin and decreased hepcidin
Besarab, 2016 [56]	60 ESA-naive incident to HD or PD	Correction	no iron oral iron iv iron	Mean ± SEM maximal change in Hb from baseline	12 weeks	Roxadustat corrected Hb levels in patients undergoing dialysis and reduced hepcidin, regardless of baseline iron status
Provenzano, 2016 [42]	90 HD (Epo alfa)	Maintenance	3:1 (epoetin alfa)	Change in Hb of ≥ 0.5 g/dL from baseline (part 1) and mean Hb level ≥ 11.0 g/dL during the last 4 treatment weeks (part 2)	19 weeks	Response rate was higher in Roxadustat than in epoetin alfa arm. Roxadustat was well tolerated
Chen, 2017 [70]	91 NDD-CKD	Correction	2:1 (placebo)	The maximum Hb change from baseline	8 weeks	Maintenance of Hb levels was reached more frequently with Roxadustat than epoetin alfa
Chen, 2017 [70]	47 DD-CKD (HD)	Maintenance	3:1 (placebo)	% of subjects with an Hb level maintained at no < 0.5 g/dL below mean baseline value	8 weeks	Maintenance of Hb levels was reached more frequently with Roxadustat than epoetin alfa

NDD, non-dialysis-dependent; DD, dialysis-dependent; CKD, chronic kidney disease; Hb, hemoglobin; ESA, erythropoiesis-stimulating agents; SEM, standard error of mean; HD, hemodialysis; PD, peritoneal dialysis.

**Table 2:** Phase 3 clinical trials of Roxadustat.

Clinicaltrials.Gov Identifier	Start/End-Date	Patients	Study Design	Intervention	Primary Endpoint
NCT02174731	June 2014/December 2019	2133 DD-CKD	Multicenter, randomized, open-label, active controlled	Roxadustat Epoetin alfa	Mean change in Hb from baseline to week 52 MACE
NCT02273726	December 2014/September 2018	741 DD-CKD	Multicenter, randomized, open-label, active controlled	Roxadustat Epoetin alfa	Hb change from baseline
NTC02174627	June 2014/December 2019	2781 ND-CKD	Multicenter, randomized, double-blind, placebo-controlled	Roxadustat Placebo	MACE
NCT02021318 (DOLOMITES)	December 2013/May 2021	616 ND-CKD	Randomized, open-label, active-controlled	Roxadustat Darbepoetin alfa	Hb response without the use of rescue therapy
NCT01887600 (ALPS)	June 2013/December 2020	594 ND-CKD	Randomized, double-blind, placebo-controlled	Roxadustat Placebo	Hb response without the use of rescue therapy
NCT02052310 (HIMALAYAS)	February 2014/September 2020	1043 incident dialysis patients	Multicenter, randomized, open-label, active-controlled	Roxadustat Epoetin alfa	Mean Hb change from baseline to week 52
NCT02278341 (PYRENEES)	October 2014/February 2021	838 DD-CKD	Randomized, open-label, active controlled	Roxadustat Epoetin alfa Darbepoetin alfa	Hb change from baseline to week 36 without rescue therapy
NCT01750190	December 2012/November 2019	922 ND-CKD	Randomized, double-blind, placebo-controlled	Roxadustat Placebo	Efficacy in anemia correction and maintenance
NCT02779764	May 2016/January 2020	164 DD-CKD	Long-Term Study	Roxadustat	Hb response rate from week 18 to week 24
NCT02780141	May 2016/January 2020	75 ESA-naives HD patient	Multicenter, randomized, 2-arm, open-label	Roxadustat	Hb response rate
NCT02780726	May 2016/December 2019	56 PD patients	Multicenter, open-label, parallel group	Roxadustat low dose, high dose and previously treated with ESA	Hb response rate from week 18 to week 24
NCT02952092	November 2016/January 2020	303 HD patients	Multicenter, randomized, 2-arm parallel, double-blind, active comparator	Roxadustat Darbepoetin alfa	Hb change from baseline to 24 week
NCT02964936	November 2016/April 2021	100 ESA-naives ND-CKD	Multicenter, randomized, 2-arm, open-label	Roxadustat	Hb change from baseline to week 24
NCT02988973	December 2016/March 2021	334 ND-CKD	Multicenter, randomized, open-label, active-comparator conversion study	Roxadustat Darbepoetin alfa	Hb change from baseline to week 24

NDD, non-dialysis dependent; DD, dialysis-dependent; CKD, chronic kidney disease; Hb, hemoglobin; ESA, erythropoiesis-stimulating agents; MACE, major cardiovascular events.
